# Regulation of arsenite oxidation by the phosphate two-component system PhoBR in *Halomonas* sp. HAL1

**DOI:** 10.3389/fmicb.2015.00923

**Published:** 2015-09-09

**Authors:** Fang Chen, Yajing Cao, Sha Wei, Yanzhi Li, Xiangyang Li, Qian Wang, Gejiao Wang

**Affiliations:** State Key Laboratory of Agricultural Microbiology, Huazhong Agricultural UniversityWuhan, China

**Keywords:** arsenite oxidation, arsenite oxidase AioBA, phosphate two-component system, regulation of gene expression, *Halomonas sp*. HAL1

## Abstract

Previously, the expression of arsenite [As(III)] oxidase genes *aioBA* was reported to be regulated by a three-component regulatory system, AioXSR, in a number of As(III)-oxidizing bacterial strains. However, the regulation mechanism is still unknown when *aioXSR* genes are absent in some As(III)-oxidizing bacterial genomes, such as in *Halomonas* sp. HAL1. In this study, transposon mutagenesis and gene knock-out mutation were performed, and two mutants, HAL1-*phoR*_931_ and HAL1-▵*phoB*, were obtained in strain HAL1. The *phoR* and *phoB* constitute a two-component system which is responsible for phosphate (Pi) acquisition and assimilation. Both of the mutants showed negative As(III)-oxidation phenotypes in low Pi condition (0.1 mM) but not under normal Pi condition (1 mM). The *phoBR* complementation strain HAL1-▵*phoB*-C reversed the mutants' null phenotypes back to wild type status. Meanwhile, *lacZ* reporter fusions using pCM-*lacZ* showed that the expression of *phoBR* and *aioBA* were both induced by As(III) but were not induced in HAL1-*phoR*_931_ and HAL1-▵*phoB*. Using 15 consensus Pho box sequences, a putative Pho box was found in the *aioBA* regulation region. PhoB was able to bind to the putative Pho box *in vivo* (bacterial one-hybrid detection) and *in vitro* (electrophoretic mobility gel shift assay), and an 18-bp binding sequence containing nine conserved bases were determined. This study provided the evidence that PhoBR regulates the expression of *aioBA* in *Halomonas* sp. HAL1 under low Pi condition. The new regulation model further implies the close metabolic connection between As and Pi.

## Introduction

Arsenic (As) is a toxic metalloid that is widely distributed in the environment and primarily exists in the inorganic forms of arsenite [As(III)] and arsenate [As(V)] (Kulp et al., 2004; Sharma and Sohn, 2009). Bacterial As(III) oxidation is an elaborate regulation process because it is not only a microbial detoxification mechanism but, in some cases, also an exergonic process that can support bacterial growth (Santini et al., 2000; Rhine et al., 2007; Kulp et al., 2008; Wang et al., 2015). The As(III) oxidase AioBA is responsible for most bacterial As(III) oxidation, and its expression was reported to be controlled by the two-component system AioSR via phosphorylation (Kashyap et al., 2006; Sardiwal et al., 2010). This regulatory model was expanded to the three-component system AioXSR in *Agrobacterium tumefaciens* 5A (Liu et al., 2012). The periplasmic As(III) binding protein AioX changes conformation after binding with As(III) and interacts with AioS, therefore involved in the regulation of As(III) oxidation with AioSR (Liu et al., 2012).

As opposed to *A. tumefaciens* 5A, in which *aioXSR* and *aioBA* are in the same operon and are transcribed in the same direction (Kang et al., 2012b; Liu et al., 2012), the *aioXSR* and *aioBA* are in different operons and are divergently transcribed in *Thiomonas arsenitoxydans* 3As (Moinier et al., 2014). The metalloprotein AioF belonging to the ArsR/SmtB family was found to be able to bind specifically to the regulatory region of the *aio* operon at two distinct positions (Moinier et al., 2014). It involved in the tightly control of the *aioBA* expression functioning together with AioXSR in *T. arsenitoxydans* 3As (Moinier et al., 2014). However, the three-component system AioXSR is absent in the genomes of some As(III)-oxidizing bacteria (e.g., *Halomonas* sp. HAL1) (Lin et al., 2012; Li et al., 2013). In these cases, the regulation mechanism responsible for the expression of *aioBA* remains unknown.

Two-component system PhoBR was reported to be responsible for the regulation of phosphate uptake and assimilation in *Escherichia coli* and other species (Wanner, 1996; Hsieh and Wanner, 2010). It is composed of a transmembrane sensory histidine kinase (HK) PhoR, and a response regulator (RR) PhoB (Hsieh and Wanner, 2010). Under phosphate-deficient conditions, PhoR catalyzes autophosphorylation on the conserved His residue, and the His-bound phosphoryl moiety is subsequently transferred to an Asp residue of PhoB. Then, the phosphorylated PhoB dimers and binds to a specific DNA sequence termed as Pho box. The DNA sequence is formed by two 7-bp direct repeats separated by a conserved 4-bp AT-rich spacer. Upon binding to the DNA, PhoB recruits the RNA polymerase and interacts with the RNA polymerase σ^70^ subunit to control the transcription of downstream genes (Makino et al., 1993; Hsieh and Wanner, 2010; Blanco et al., 2011, 2012). In *E. coli*, 31 genes in nine transcripts (*eda, phnCDEFGHIJKLMNOP, phoA, phoBR, phoE, phoH, psiE, pstSCAB*-*phoU*, and *ugpBAECQ*) were directly controlled by PhoBR (Hsieh and Wanner, 2010). In addition, PhoBR was reported to regulate the expression of genes related to chemotaxis, antibiotic resistance, and virulence attenuation (Pratt et al., 2009; Crepin et al., 2011; Srinivasan et al., 2012), indicating that PhoBR plays a key role in a variety of bacterial physiological metabolisms. Previously, it was reported that the phosphate (Pi) stress-response genes *phoB, pstS*, and *phoU* are intricately co-regulated with As(III) oxidation genes in *A. tumefaciens* 5A (Kang et al., 2012b), indicating PhoBR may involve in the regulation of As(III) oxidation.

In this study, we developed *Halomonas* sp. HAL1 as a model for understanding the regulation of As(III) oxidation in which AioXSR is absent. Using transposon mutagenesis, gene knock-out mutation and complementation, we found that *phoR* and *phoB* both influenced As(III) oxidation. Because the Pi concentration is reported to be associated with the expression of the As(III) oxidase AioBA (Kang et al., 2012b), both low and normal Pi conditions are used to conduct the regulation analysis in this study. Bacterial one-hybrid system and electrophoretic mobility shift assay (EMSA) were employed to demonstrate that PhoB could bind with the putative Pho box in the regulatory region of *aioBA*. The results of this study indicated that the two-component system PhoBR can regulate the As(III) oxidation in *Halomonas* sp. HAL1 under the low Pi condition. The results of this study revealed a novel regulation mechanism of bacterial As(III) oxidation, supplemented the co-regulation mechanism between As and Pi.

## Results

### The *phoBR* system effected on As(III) oxidation

**Figure 1:**
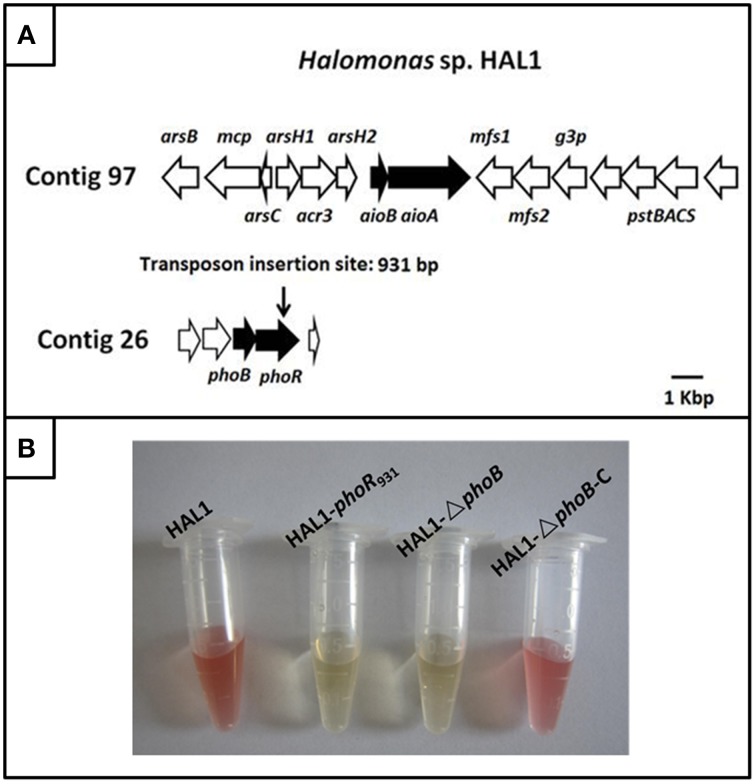
**The gene island of As(III) oxidation in ***Halomonas*** sp. HAL1 and As(III) oxidation analysis. (A)** The gene island of As(III) oxidation. The transposon insertion site of *phoR* mutant is shown by vertical arrow. **(B)**. As(III) oxidation phenotype of strains HAL1, HAL1-*phoR*_931_, HAL1-Δ*phoB*, and HAL1-Δ*phoB*-C. The strains were inoculated into MMNH_4_ medium containing 0.1 mM Pi, 0.8 M NaCl, and 1 mM As(III). After 7 d cultivation, the As(III) oxidation was monitored by qualitative KMnO_4_ biochemical analysis.

The draft genome sequencing of *Halomonas* sp. HAL1 revealed that an arsenic gene island located in contig 97 contains the As(III) oxidase genes *aioBA*, the arsenic efflux genes *arsB*-*mcp*-*arsC*-*arsH1*-*acr3*-*arsH2* and the phosphate-related genes *pstSCAB* (Figure 1A) (Lin et al., 2012). However, the three-component regulator genes *aioXSR* were absent in the entire genome (Lin et al., 2012; Li et al., 2013). Genomic analysis revealed that only one copy of *phoBR* genes locating in contig 26. The two genes encode two-component system proteins, which is mainly a response to phosphate stress (Lin et al., 2012; Li et al., 2013).

To identify the putative genes related to As(III) oxidation, we used transposon mutagenesis in combination with qualitative KMnO_4_ screening to isolate the As(III) oxidation mutants. Five mutants disabled for As(III) oxidation were generated by screening approximately 2000 tranformates; these five mutants were interrupted in the genes encoding molybdenum cofactors (two mutants), cytochrome c, As(III) oxidase, and PhoR. All of the proteins were reported to be correlated with bacterial As(III) oxidation (Rosen, 1995; Silver and Phung, 2005; Kang et al., 2012b). We paid particular attention to PhoR because we were more interested in the regulation mechanisms of As(III) oxidation. Due to PhoB is the response regulator in the two-component system PhoBR, the mutant HAL1-▵*phoB* and the complementary strain HAL1-▵*phoB*-C were then constructed using the allelic exchange vector pCM184 and vector pCT-zori (broad-host-range, *lacZ*α selection marker) constructed in this study (Figure S1). The qualitative KMnO_4_ tests indicated that the disruption of *phoR* or *phoB* deprived the As(III) oxidation ability in *Halomonas* sp. HAL1, while the complementary strain recovered the phenotype back to the wild type strain (Figure 1B).

**Figure 2:**
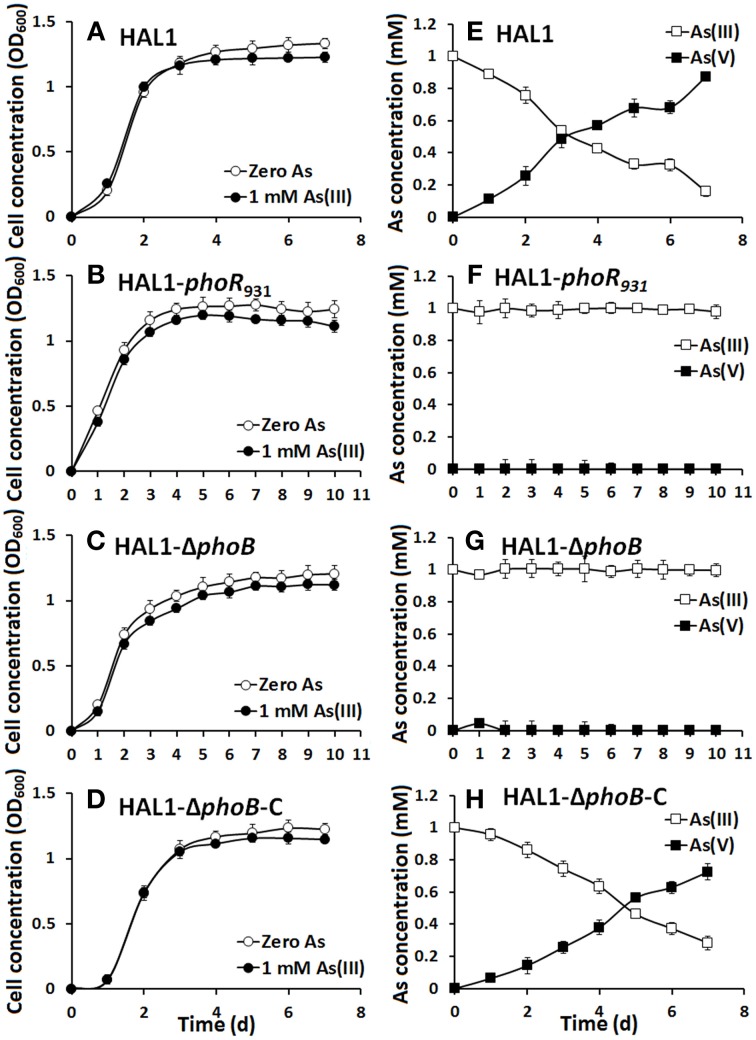
**As(III) oxidation was influenced by ***phoBR*** in low Pi condition. (A–D)** The growth curves of strains HAL1, HAL1-*phoR*_931_, HAL1-Δ*phoB*, and HAL1-Δ*phoB*-C in MMNH_4_ medium containing 0.1 mM Pi and 0.8 M NaCl, with or without the addition of 1 mM As(III). **(E–H)** As(III) oxidation profiles of the same strains. As(III) and As(V) concentrations in the cultural fluids were measured using HPLC-HG-AFS. Data symbols shown in **(A)** are the same for **(B–D)**, and data symbols shown in **(E)** are the same for **(F–H)**. Data are shown as the mean of three replicates, with the error bars illustrating one standard deviation.

**Figure 3:**
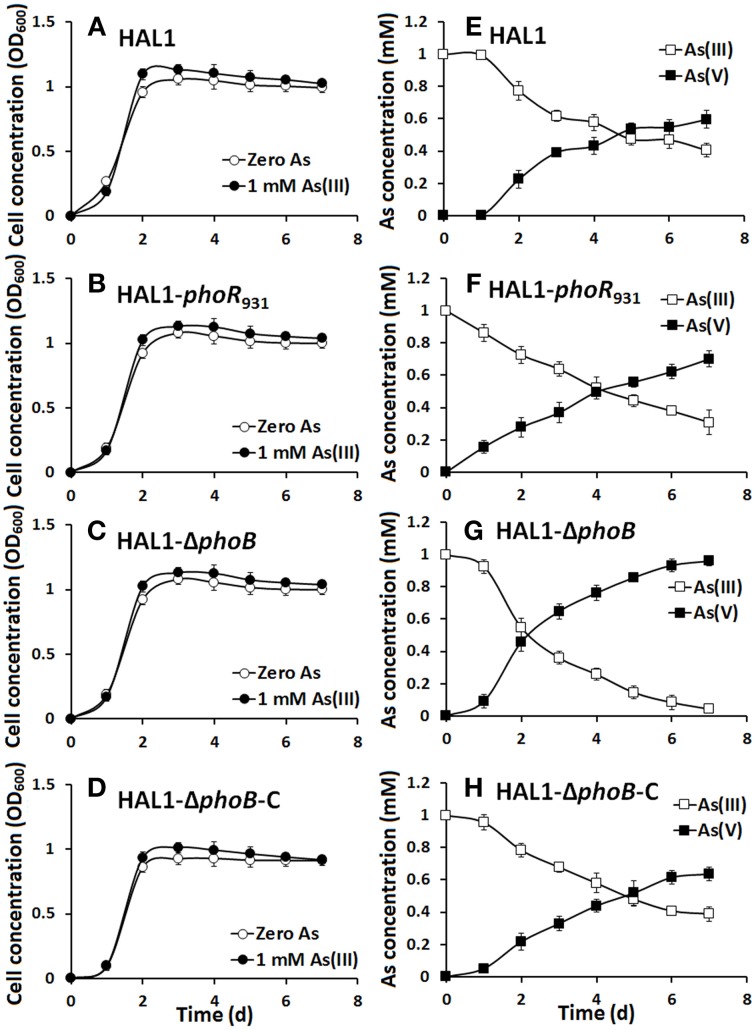
**As(III) oxidation was not influenced by ***phoBR*** in normal Pi condition. (A–D)** The growth curves of strains HAL1, HAL1-*phoR*_931_, HAL1-Δ*phoB*, and HAL1-Δ*phoB*-C in MMNH_4_ medium with the presence of 1 mM Pi and 0.8 M NaCl, with or without the addition of 1 mM As(III). **(E–H)** As(III) oxidation profiles of the same strains. As(III) and As(V) concentrations in the cultural fluids were measured using HPLC-HG-AFS. Data symbols shown in **(A)** are the same for **(B–D)**, and data symbols shown in **(E)** are the same for **(F–H)**. Data are shown as the mean of three replicates, with the error bars illustrating one standard deviation.

The growth curves and As(III) oxidation efficiencies of the *Halomonas* sp. strains were tested in both low Pi (0.1 mM) and normal Pi (1 mM) conditions (Figures 2, 3). Consistent with the qualitative KMnO_4_ tests, the As(III) oxidation phenotypes disappeared at the low Pi condition in the *phoR* or *phoB* disruption strains, while the complemented strain HAL1-Δ*phoB*-C reversed the mutants null phenotype back to wild type status (Figure 2). However, in the normal Pi condition, all of the four *Halomonas* sp. strains showed similar As(III) oxidation rates during the 7-day test (Figure 3), indicating that PhoBR may only have an effect on As(III) oxidation at the low Pi condition. The results are consistent with the properties of PhoBR because they are the regulators responsible for Pi stress (Hsieh and Wanner, 2010).

### phoBR influenced the expression of *aioBA*

**Figure 4:**
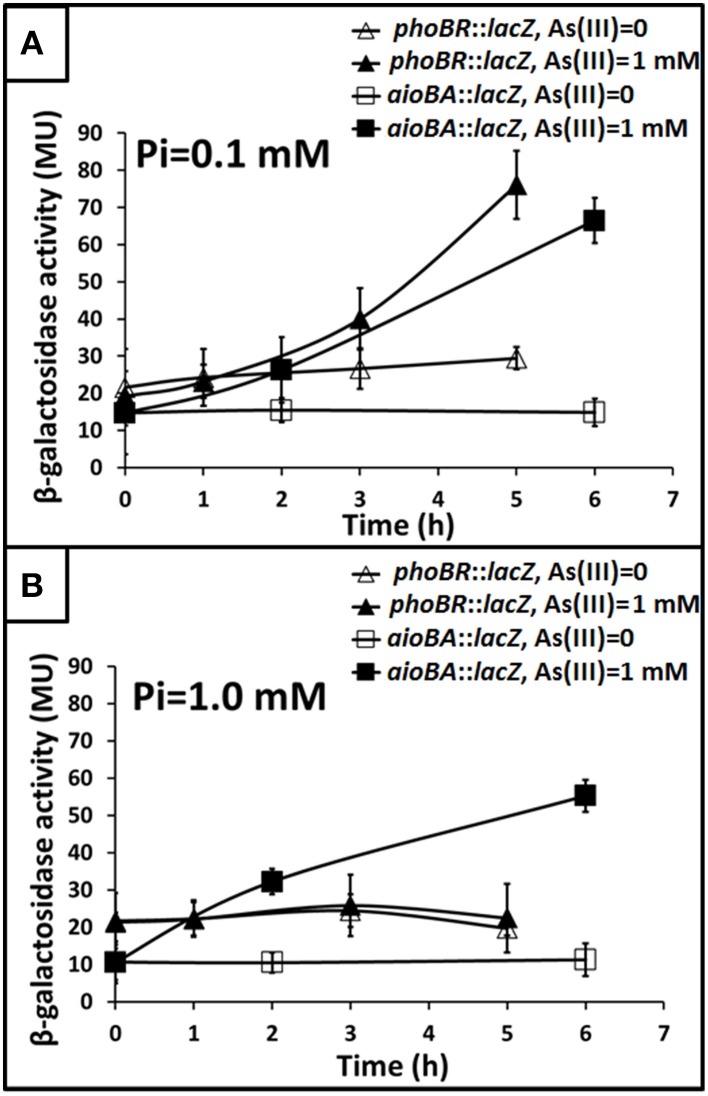
**Influence of As(III) on the expression of ***phoBR*** and ***aioBA*****. Expression of *phoBR::lacZ* and *aioBA::lacZ* in strain HAL1 were monitored. Data are shown as the mean of three replicates, with the error bars illustrating one standard deviation. **(A)** Bacteria were cultured in MMNH_4_ medium containing 0.1 mM Pi and 0.8 M NaCl, with or without the addition of 1 mM As(III). **(B)** Bacteria were cultured in MMNH_4_ medium containing 1 mM Pi and 0.8 M NaCl, with or without the addition of 1 mM As(III).

**Figure 5:**
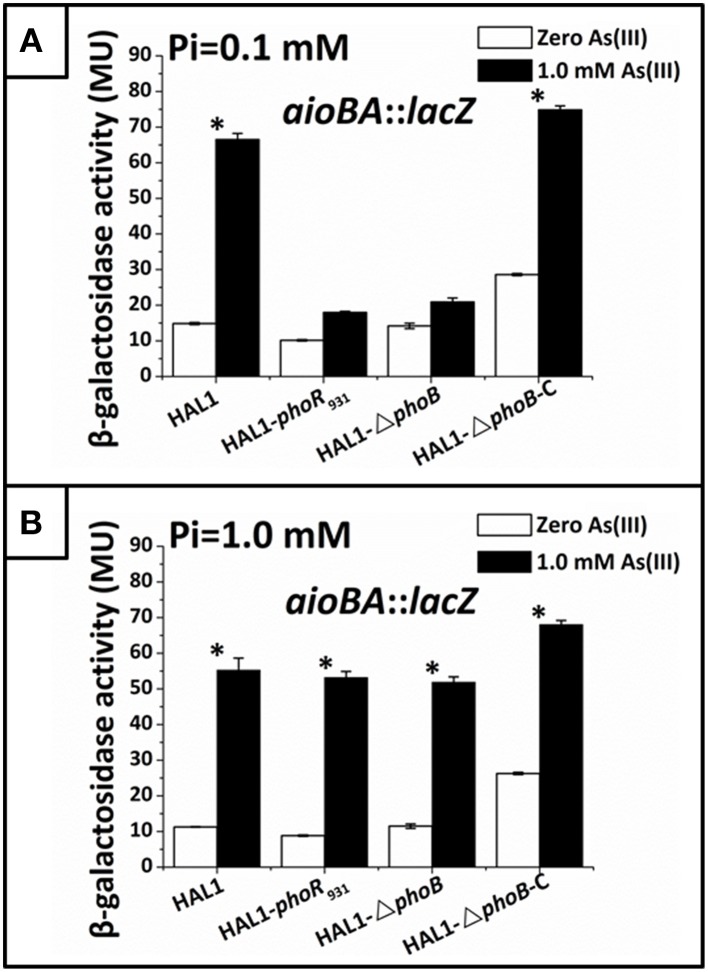
**Quantitative ***lacZ*** reporter gene analysis of ***aioBA::lacZ*** expression in strains HAL1, HAL1-***phoR***_931_, HAL1-Δ***phoB***, and HAL1-Δ***phoB***-C**. β-galactosidase activity is presented as Miller units. Data are shown as the mean of three replicates, with the error bars represent ± 1 SD. **(A)** Bacteria were cultured in MMNH_4_ medium containing 0.1 mM Pi and 0.8 M NaCl, with or without the addition of 1 mM As(III). With the addition of As(III), the mean values of strain HAL1 and HAL1-▵*phoB*-C were significantly different from the ones with the absence of As(III) (^*^p < 0.05). **(B)** Bacteria were cultured in MMNH_4_ medium containing 1 mM Pi and 0.8 M NaCl, with or without the addition of 1 mM As(III). With the addition of As(III), the mean values of all the four strains were significantly different from the ones with the absence of As(III) (^*^p < 0.05).

To understand how *phoBR* and *aioBA* respond to As(III) in *Halomonas* sp. HAL1, quantitative *lacZ* reporter gene analyses were performed with the *lacZ* reporter vector pCM-*lacZ* constructed in this study (Figure S2). The expressions of *phoBR*::*lacZ* and *aioBA*::*lacZ* were not induced (Figure 4A) in 5− or 6-h cultivations in the low Pi condition without As(III). However, in the low Pi condition with the addition of 1 mM As(III), the expressions of *phoBR*::*lacZ* and *aioBA*::*lacZ* increased with increasing induction time, and the *phoBR*::*lacZ* expression appeared to be higher than that of *aioBA*::*lacZ* (Figure 4A). Meanwhile, in the low Pi condition, the expression of *aioBA*::*lacZ* was significantly induced by As(III) after 6 h of cultivation in strains HAL1 and HAL1-Δ*phoB*-C, but no statistically significant inductions in the mutants HAL1-*phoR*_931_ and HAL1-Δ*phoB* were observed (Figure 5A). However, in the normal Pi condition, the expression levels of *phoBR*::*lacZ* were similar in the presence or absence of As(III), and the expression of *aioBA*::*lacZ* was again significantly induced by As(III) (Figure 4B). In addition, the disruption of *phoB* and *phoR* had no effect on the expression of *aioBA*::*lacZ* in the normal Pi condition (Figure 5B). The expression of *aioBA* in low Pi and normal Pi conditions were consistent with the As(III) oxidation phenotypes (Figures 2, 3). The above results indicated that PhoBR regulates the expression of *aioBA* in low Pi condition but not in normal Pi condition.

### PhoB binds to the regulatory region of *aioBA*

**Figure 6:**
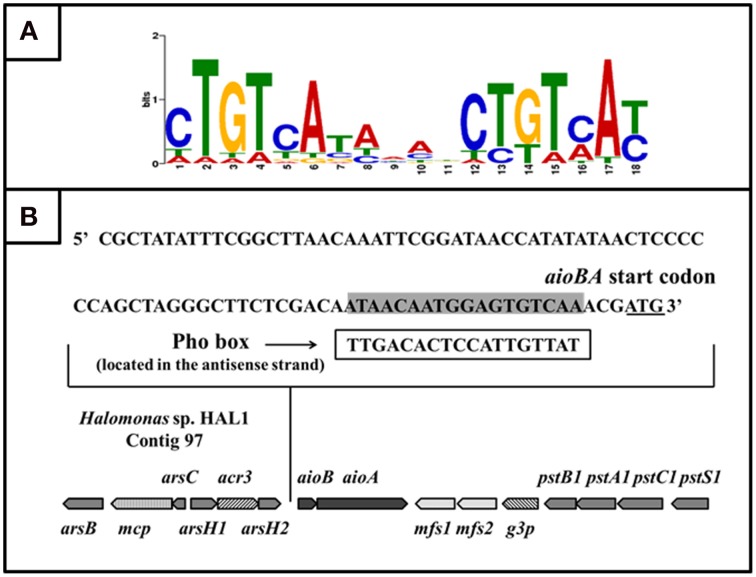
**The putative PhoB binding site (Pho box) in the ***aioBA*** promoter region. (A)** Sequence logo of Pho motif predicted by MEME program based on the known Pho boxes. Higher bit scores indicate more conservation at respective site. **(B)** The putative Pho box sequence was denoted by rectangular box, which is reverse complement with the sequence in sense strand (Labeled gray).

Based on 15 reported Pho box sequences (Yuan et al., 2006), we predicted a Pho motif using the MEME on-line program (http://meme.nbcr.net/meme/cgi-bin/meme.cgi; Bailey and Elkan, 1994; Figure 6A). With the predicted Pho motif, we found a putative Pho box (TTGACACTCCATTGTTAT) located in the antisense strand within −21 to −3 bp upstream from the *aioB* start codon (Figure 6B); this box is 56% identical to the *E. coli* consensus Pho box (Diniz et al., 2011).

**Figure 7:**
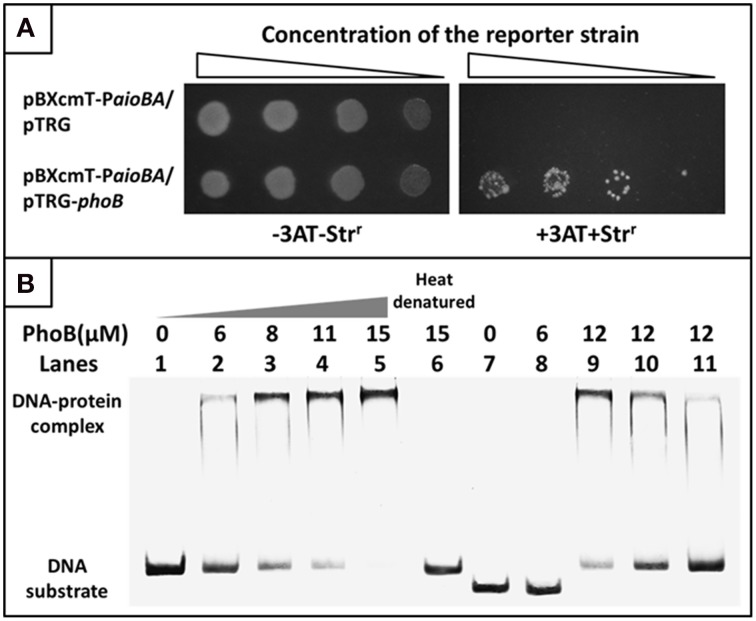
**Bacterial one-hybrid system and EMSA analyses for the interaction between PhoB and the ***aioBA*** promoter region. (A)** Bacterial one-hybrid assay. A co-transformant containing vector pTRG and pBXcmT-P*aioBA* was used as the self-activation control. −3AT-Str^r^ represents the LB plate, +3AT+Str^r^ represents the screening plate. **(B)** EMSA assays. Lanes 1–6, 1.6 pmol FAM-labeled *aioBA* regulatory region DNA were co-incubated with various amounts of PhoB activated or heat-denatured; Lanes 7–8, 1.6 pmol FAM-labeled DNA sequence without putative Pho box were co-incubated with PhoB; Lanes 9–11, the competition assay using 1.6 pmol FAM-labeled *aioBA* regulatory region DNA and 12 μM PhoB against 1.4, 2.7, and 6.1 pmol unlabeled *aioBA* regulatory region DNA.

To examine the interaction between PhoB and the putative Pho box, we first used a bacterial one-hybrid system to test the protein-DNA interaction based on the transcriptional activation of *HIS3* and *aadA* (Guo et al., 2009). The regulatory sequence of *aioBA* containing the putative Pho box was cloned into *HIS3*-*aadA* upstream of the reporter vector pBXcmT, while the PhoB coding region was introduced into pTRG vector. Both of the two constructed vectors were then transferred into a reporter strain. The reporter strain containing pBXcmT-Paio*BA* and pTRG-*phoB* grew well on the screening plate containing 3-AT and Str, and the negative control (the reporter strain containing pBXcmT-Paio*BA* and pTRG) did not grow, which indicated that PhoB could interact with the *aioBA* regulatory region *in vivo* (Figure 7A).

A purified His_6_-tag PhoB (Figure S3) and a 209-bp *aioBA* regulatory sequence containing the putative Pho box were then used to test the interaction between PhoB and the putative Pho box *in vitro* using EMSA. With increasing PhoB concentration, the free DNA substrates gradually disappeared, while the intensity of the shifted DNA band increased (Figure 7B). Neither heat-denatured PhoB nor non-specific DNA (excluding the putative Pho box) exhibited a retardation band in the EMSA gel (Figure 7B). Moreover, the unlabeled DNA substrate could competitively inhibit the binding of PhoB to the labeled DNA substrate (Figure 7B). The results indicated that PhoB could bind specifically to the *aioBA* regulatory region.

**Figure 8:**
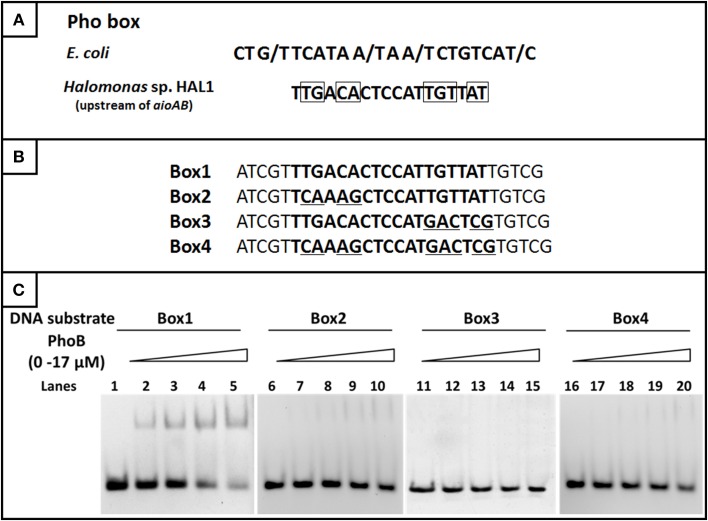
**EMSA for identifying the binding sites in the ***aioBA*** promoter region. (A)** The comparison of the consensus Pho box in *E. coli* and the putative Pho box in the *aioBA* regulatory region. The optional bases were marked by slash, while the conserved bases in the Pho box of *aioBA* were marked with rectangle. **(B)** The four tested DNA substrates. All of the tested binding sequences were shown in bold. Box1 is the original Pho box in the *aioBA* regulatory region. Box2, Box3, and Box4 were mutated in different conserved bases. The mutation base pairs were marked with underline. **(C)** EMSA for the DNA-binding activity of PhoB with different substrates. Each of four oligonucleotide substrates was mixed with 0–17 μM PhoB. PhoB could bind with Box1, but not with Box2, Box3, or Box4 which were mutated in the conserved sites.

Based on the comparison of the Pho boxes between *E. coli* and the regulatory region of the *aioBA* genes, the conserved base pairs in the 18 bp Pho box were predicted (Figure 8A). To test the exact binding sequence of PhoB in the regulatory region of *aioBA* genes, a short DNA fragment containing the putative Pho box (18 bp) and its bilateral five bases (protection bases) were used for EMSA testing (Box1, Figure 8B), and the conserved nine base pairs were site-directed substituted separately, generating Box2, Box3, and Box4 (Figure 8B). As shown in Figure 8C, PhoB was capable of binding with substrates containing the putative Pho box (Box1), but was incapable of binding with the mutated sequences Box2, Box3, and Box4. This indicated that the 18-bp putative Pho box (TTGACACTCCATTGTTAT) is essential for PhoB to bind with the *aioBA* regulatory region, and the nine conserved bases (Figure 8A) are the critical recognition sites.

## Discussion

Currently, the regulation models of As(III) oxidation have only been reported in the As(III)-oxidizing strains that contain *aioXSR* in their genomes (Kang et al., 2012a,b; Liu et al., 2012; Li et al., 2013; Moinier et al., 2014). However, the potential proteins involved in the regulation of As(III) oxidase genes in As(III)-oxidizing strains without *aioXSR*, such as *Halomonas* sp. HAL1 (Lin et al., 2012), remain unknown. In this study, after discovering that the *phoR* mutant affected the As(III) oxidation phenotypes by transposon mutagenesis, we predicted that PhoBR may regulate the expression of As(III) oxidase genes in strain HAL1 because PhoBR can activate/inhibit the transcription of numerous genes (Yuan et al., 2006; Lamarche et al., 2008; Hsieh and Wanner, 2010).

Subsequently, we conducted a comprehensive analysis to assess this hypothesis. We concluded that PhoBR could regulate the expression of *aioBA* under low-Pi condition based on the following observations: (i) the mutants of *phoR* or *phoB* affected the As(III) resistance and oxidation phenotypes under low Pi condition; (ii) PhoBR influenced the expression of *aioBA*; (iii) As(III) induced the expression of PhoBR; and (iv) PhoB could bind with the putative pho box located in the regulation region of *aioBA*.

We also found that low Pi levels increased the As(III) oxidation efficiency in strain HAL1 and is consistent with the report showing that bacterial As(III) oxidation is more efficient in low Pi condition than in normal or high-Pi conditions (Kang et al., 2012b; Wang et al., 2015). In *A. tumefaciens* that containing AioXSR, the Pi stress response genes (*phoB, pstS*, and *phoU*) are intricately co-regulated with the As(III) oxidation genes *aioBA*, and the regulatory cross-talk via phosphorelay to PhoB from AioS has been proposed (Kang et al., 2012b), in which AioXSR plays the dominant role in As(III) oxidation. Although the Pi stress response proteins are related to As(III) oxidation, the regulatory relationship between PhoBR and As(III) oxidation appeared to be indirect in strain 5A, since no recognizable Pho box was found in the *aioSRBA* regulation region (Kang et al., 2012b). In this study, the *aioXSR* are absent in strain HAL1, but the low Pi concentration still showed effect on As(III) oxidation and the expression of *phoB* was also induced by As(III), similar to *A. tumefaciens* 5A (Kang et al., 2012b). These results imply that As and Pi metabolisms are also likely to be co-regulated in strain HAL1. However, the co-regulation mechanisms should be different between the strains HAL1 and 5A, since the suggested cross-talk phosphorylation between the AioXSR and PhoRB (Kang et al., 2012b) cannot be exist in strain HAL1. In addition, since strain HAL1 does not carry *aioXSR*, the co-regulation between As and Pi are more likely related to the concentrations As and Pi which needs to be further investigated.

Even though the PhoBR was reported in numerous studies to respond to Pi stress, the PhoB or PhoR have never showed to bind Pi directly, instead, PstS was the protein to bind Pi, As(V) (Hsieh and Wanner, 2010; Wang et al., 2015), and possibly As(III). In some situations, the two-component system does not response environmental signals directly. For example, in our previous study with *A. tuemfaciens* 5A, even though the As(III)-response two-component system AioSR regulates the expression of As(III) oxidase genes *aioBA*, there is no As(III) binding domain in AioS or AioR. Instead, a protein called AioX binds As(III) directly (Liu et al., 2012), and AioX possibly transfers the As(III) signal to AioS. In our study, it is also possible that the “PstS” has the ability to sense Pi or As(III) and transfer the signals to PhoR in strain HAL1. PhoR auto-phosphorylates and transfers Pi group to PhoB, which then binds to the *aioBA* promoter region containing a putative Pho box and transcriptionally activates the expression of *aioBA* in an As(III)-dependent manner.

Based on the results of this study, the regulation of As(III) oxidation in different As(III)-oxidizing strains can be classified into three models: (i) the regulation model of the three-component system, AioXSR (Liu et al., 2012); (ii) the co-regulation model of AioXSR with the metalloprotein AioF belonging to the ArsR/SmtB family (Moinier et al., 2014); and (iii) the regulation model manipulated by a two-component system, PhoBR (this study). Since the As(III)-oxidizing strains that lack *aioXSR* genes were reported increasingly (Cai et al., 2009; Osborne et al., 2013; Koechler et al., 2015), the PhoBR regulation model may be widespread in these As(III)-oxidizing strains. Pi is generally present at low concentrations in terrestrial ecosystems (Vieira et al., 2008), thus the regulation of *aioBA* expression by PhoBR is reasonable when AioXSR is absent in As(III)-oxidizing strains. Moreover, it is obvious that As(III) oxidation was unaffected by the mutation of *phoB* or *phoR* under normal Pi conditions. Since Pit system was reported to involve in the Pi acquisition under normal Pi conditions (Hsieh and Wanner, 2010), it is very likely that the regulation models are different between low and normal Pi conditions.

## Materials and methods

### Bacterial strains and growth conditions

The bacterial strains and plasmids used for this study are listed in Table S1, while the primers are listed in Table S2. *Halomonas* sp. HAL1 and its derivative strains were cultured at 28°C in a defined minimal mannitol medium (MMNH_4_) (Somerville and Kahn, 1983) containing 0.1 mM or 1 mM Pi (K_2_HPO_4_·3H_2_O:KH_2_PO_4_ = 4:1, pH = 7.2) and 0.8 M NaCl. When needed, 1 mM NaAsO_2_ [As(III)] was added into the medium. *E. coli* strains were grown at 37°C in Luria-Bertani (LB) medium. Stock solutions of kanamycin (Kan, 50 mg/mL), chloramphenicol (Cm, 25 mg/mL), tetracycline (Tet, 5 mg/mL), and streptomycin (Str, 8 mg/mL) were added when required.

### Transposon mutagenesis in combination with qualitative KMnO_4_ screening to isolate arsenite oxidation mutant

A Kan resistance transposon delivery vector, pRL27, encoding a hyperactive Tn5 transposase was used to construct a transposon mutant library in this study (Larsen et al., 2002). *E. coli* strain S17-1 was employed to transfer pRL27 into the recipient strain *Halomonas* sp. HAL1. The transformants were selected on MMNH_4_ agar plates (Somerville and Kahn, 1983) containing 0.1 mM Pi and 0.8 M NaCl with 50 μg/mL Kan. After 4 d cultivation at 28°C, the colonies were inoculated into the same liquid medium in a 96-well plate containing 0.1 mM Pi, 0.8 M NaCl, 50 μg/mL Kan, and 200 μM As(III). Followed by 7 d cultivation at 28°C with shaking at 100 rpm, qualitative KMnO_4_ screening was used to detect the As(III) oxidation phenotypes of transformants (Fan et al., 2008). Using the As(III)-oxidizing strain HAL1 as a positive control, the candidate mutant strains were screened based on the yellow color tested with KMnO_4_, which indicates the disability of As(III) oxidation. Then, DNA of each potential mutant was extracted, digested with *Bam*HI (Fermentas) and self-ligated using T4 DNA ligase (Promega) before being transferred into *E. coli* strain S17-1. The transformants were selected on LB agar plates containing 50 μg/mL Kan, and then after 12–24 h of cultivation at 37°C, the plasmid of each transformant was extracted. The primers pRLSR and pRLSF (Table S2) were used to amplify the flanking sequences of Tn5 transposon by inverse PCR. After the sequencing, the insertion sites were analyzed using the NCBI BLAST server (http://blast.ncbi.nlm.nih.gov/Blast.cgi) based on the genome of strain HAL1 (Lin et al., 2012).

### Construction of *phoB* mutant and the complemented strains

The suicide allelic exchange vector pCM184 was used to construct the *phoB* mutant in *Halomonas* sp. HAL1 (Marx and Lidstrom, 2002). The bilateral flanking regions of *phoB* were amplified by PCR using the primers phoB-up-F/phoB-up-R and phoB-down-F/phoB-down-R (Table S2). The upstream PCR fragment was then cloned into *Aat*II-*Bsr*GI sites, while the downstream PCR fragment was introduced into the *Sac*II-*Sac*I sites of pCM184. The resulting *phoB* allelic exchange vector pCM184-BUD was then mobilized into strain HAL1 via conjugation with *E. coli* strain S17-1, and the double crossing over *phoB* mutants were selected using 50 μg/mL Kan. Followed by screening with 25 μg/mL Tet, the Tet-sensitive and Kan-resistant mutants were identified by PCR using the primers phoB-upYZ-F/phoB-upYZ-R and phoB-downYZ-F/phoB-downYZ-R (Table S2).

A broad host vector pCT-Zori was constructed (see the Supplementary Materials) and used to generate the complementary strain of *phoB* mutant. For Δ*phoB* complementation, the complete *phoBR* coding region along with the 450-bp upstream regulatory region was PCR amplified and cloned into the *Sac*I and *Hin*dIII sites of pCT-zori, resulting in pCT-zori-*phoBR*. This plasmid was transformed into *E. coli* strain S17-1 and conjugated with strain HAL1-▵*phoB*. The complementary strain HAL1-▵*phoB*-C was selected by Cm resistance and confirmed by PCR using primers PhoB-HB-F/ PhoB-HB-R (Table S2) along with sequencing.

### Growth and As(III) oxidation tests

The cultures of HAL1, HAL1-*phoR*_931_, HAL1-▵*phoB*, and HAL1-▵*phoB*-C (OD_600_ ~ 0.5) were each inoculated (1 mL) into 100 mL MMNH_4_ containing 0.1 mM or 1 mM Pi and 0.8 M NaCl in the presence or absence of 1 mM As(III). They were then cultured at 28°C by shaking at 160 rpm for up to 10 d. Each day, culture samples were taken for monitoring optical density (OD_600_) using a spectrophotometer (Beckman DU800; Beckman, Fullerton, CA) and quantifying As(III) and As(V) with a combination of high-performance liquid chromatography with hydride-generation atomic fluorescence spectroscopy (HPLC-HG-AFS) (Beijing Titan Instruments Co., Ltd., China), as previously described (Liao et al., 2013).

### Reporter gene assays of *aioBA* and *phoBR*

The reporter gene assays in this study was tested by β-galactosidase activity (Miller, 1972). The promoter regions of *aioBA* and *phoBR* were amplified from strain HAL1 (primers listed in Table S2) and introduced into the *Bgl*II-*Bsr*GI sites of pCM-*lacZ* (see the Supplementary Materials), respectively. The resulting plasmids were then introduced into *Halomonas* sp. strains via biparental conjugation. When the cell OD_600_ reached approximately 0.4, the cells were harvested by centrifugation (8000 rpm, 10 min) and inoculated with the same OD_600_ into the MMNH_4_ medium containing 0.8 M NaCl and 1 mM As(III) along with the addition of 0.1 mM or 1 mM Pi. The cultures without As(III) were used as the negative controls. All of the cultures were cultured at 28°C on a rotary shaker. During the incubation, β-galactosidase assays were conducted as described previously (Miller, 1972; Kang et al., 2012b).

### Bacterial one-hybrid system assay

The Pho box-PhoB interaction was tested *in vivo* using a bacterial one-hybrid system as described previously (Guo et al., 2009). The *phoB* coding region was amplified (primers listed in Table S2) and cloned into the *Bam*HI-*Eco*RI sites of the pTRG vector (Stratagene), generating pTRG-*phoB*. The regulatory region of *aioBA* including the putative Pho box was amplified (primers listed in Table S2) and inserted directly into the *Xcm*I site of pBXcmT (Guo et al., 2009), yielding pBXcmT-P*aioBA*. Both of the two recombinant plasmids were co-transformed into the reporter strain *E. coli* XL1-Blue MRF' Kan (Stratagene). After 3–4 d of cultivation at 28°C on a selective screening medium plate containing 20 mM 3-amino-1,2,4-triazole (3-AT), 16 μg/mL Str, 15 μg/mL Tet, 34 μg/mL Cm, and 50 μg/mL Kan, the co-transformant growth was tested (Guo et al., 2009). In addition, a co-transformant containing vector pTRG and pBXcmT-P*aioBA* was used as the self-activation control.

### Electrophoretic mobility shift assay (EMSA)

To identify the putative Pho box in the *aioBA* regulatory region, a 209-bp fragment containing the putative Pho box of the *aioBA* regulatory region was amplified using PaioA-F/PaioA-R (Table S2). The primer PaioA-F was labeled with fluorophore FAM when needed. EMSA was carried out with a 1.6-pmol labeled probe and increasing concentrations of PhoB (from 0 to 15 μM), while the heat-denatured PhoB and unspecific DNA (exclude putative Pho box) were used as negative controls. For competition assay, 1.4-, 2.7-, and 6.1-pmol unlabeled probes were added to reaction mixtures containing 12 μM PhoB and the 1.6-pmol labeled probe. All reaction mixtures were incubated at 4°C for 30 min in binding buffer [20 mM Tris-HCl, pH 7.0; 50 mM NaCl; 1 mM dithiothreitol (DTT); 10 mM MgCl_2_; 100 μg/ml bovine serum albumin (BSA)] and then loaded onto a 4% native PAGE. Followed by 2 h of running at 100 V in 0.5 × TBE buffer, gels were exposed to a phosphor imaging system (Fujifilm FLA-5100).

To test the exact binding sequence of PhoB in the regulatory region of *aioBA* genes, a 28-bp DNA fragments containing an 18 bp putative Pho box and bilateral 5-bp protection bases, were synthesized by Tsingke (Tsingke Biological Technology Company, Beijing, China) and directly annealed *in vitro*. EMSA for the DNA binding activity of PhoB was carried out on different substrates using 14.5-pmol probes with increasing concentrations of PhoB (0–17 μM).

## Author contributions

FC designed and performed the experiments and wrote the manuscript. YC, SW, YL, and XL participated in the experiments. QW wrote and revised the draft of the manuscript. GW designed the study and revised the draft of the manuscript. All authors read and approved the final manuscript.

### Conflict of interest statement

The authors declare that the research was conducted in the absence of any commercial or financial relationships that could be construed as a potential conflict of interest.
