# Supplementary material for: Regulation of arsenite oxidation by the phosphate two-component system PhoBR in Halomonas sp. HAL1
Source: Front Microbiol. 2015 Sep 9;6:923. doi: 10.3389/fmicb.2015.00923 (PMC4563254; doi:10.3389/fmicb.2015.00923)
Supplement: Supplementary file 1 [file Table1.DOCX]

**Table S1 The strains and plasmids used in this study.**

| **Strain/plasmid** | **Relevant properties or derivation** | **Source or reference** |
| --- | --- | --- |
| **Strains** |  |  |
| ***Halomonas* sp.** |  |  |
| HAL1 | Wild type, As(III) oxidizing, Rif^r^ | Lab stock |
| HAL1-*phoR*_931_ | *phoR* Tn5 insertion mutant | This study |
| HAL1-△*phoB* | *phoB* replaced by Km mutant | This study |
| HAL1-△*phoB*-C | HAL1-△*phoB* complement with *phoBR* genes | This study |
| BL21-*phoB* | PhoB expression strain, Km^r^ | This study |
| XL1-Blue  (pTRG-*phoB*, pBXcmT-P*aioBA*) | Bacterial one-hybrid reporter strain, Km^r^, Tet^r^, Cm^r^ | This study |
| HAL1 (*aioBA::lacZ*) | *aioBA* promoter activity of HAL1 reporter strain, Cm^r^ | This study |
| HAL1-△*phoB* (*aioBA::lacZ*) | *aioBA* promoter activity of HAL1-△*phoB* reporter strain, Km^r^, Cm^r^ | This study |
| HAL1-*phoR*_931_ (*aioBA::lacZ*) | *aioBA* promoter activity of HAL1-*phoR*_931_ reporter strain, Km^r^, Cm^r^ | This study |
| HAL1-△*phoB*-C (*aioBA::lacZ*) | *aioBA* promoter activity of HAL1-△*phoB*-C reporter strain, Km^r^, Cm^r^ | This study |
| HAL1 (*phoBR::lacZ*) | *phoBR* promoter activity of HAL1 reporter strain, Cm^r^ | This study |
| ***Escherichia coli*** |  |  |
| S17-1 | *recA hsdR thi pro* Sm^R^ RP4-2-TcR:: Mu-Km:: Tn7 | (Simon et al., 1983) |
| BL21 | B F– *dcm ompT hsdS*(r_B_^-^m_B_^-^) *gal* λ(DE3) | Novagen |
| XL1-Blue | △(mcrA)183△(mcrCB-hsdSMR-mrr)173 endA1 supE44 thi-1 recA1 gyrA96 relA1lac [F’ proAB lacIqZ△M15 Tn5 Km^r^] | Stratagene |
| **Plasmids** |  |  |
| pRL27 | Transposon vector , *ori*R6K, Km^r^ | (Larsen et al., 2002) |
| pCM184 | Ap^r^, Km^r^, Tc^r^, oriT double exchange vector | (Marx and Lidstrom, 2002) |
| pCM184-BUD | pCM184 containing *phoB* upstream and downstream fragments | This study |
| pBHR1 | Km^r^, Cm^r^, oriT broad host vector | (Szpirer et al., 2001) |
| pGEM-4Z | Ap^r^, subcloning vector | Promega |
| pCT-zori | Cm^r^, oriT broad host vector | Lab stock |
| pCT-zori-*phoBR* | *phoBR* genes with its promoter cloned into the MCS of pCT-Zori | This study |
| pET28a | Km^r^, His6 Tag expression vector | Novagen |
| pET28a-*phoB* | *phoB* in frame fusion to the multiple sites of pET-28a | This study |
| pTRG | Tet^r^, for bacterial one-hybrid assay | Stratagene |
| pTRG-*phoB* | pTRG containing *phoB* coding region | This study |
| pBXcmT | Cm^r^, for bacterial one-hybrid assay | (Guo et al., 2009) |
| pBXcmT-P*aioBA* | pBXcmT containing *aioBA* promoter region | This study |
| pCM184-Cm | Km of pCM184 was replaced by Cm | This study |
| pCM-*lacZ* | pCM184-Cm containing *lacZ* coding region | This study |
| pCM-*lacZ*-P*aioBA* | pCM-*lacZ* containing *aioBA* promoter region | This study |
| pCM-*lacZ*-P*phoBR* | pCM-*lacZ* containing *phoBR* promoter region | This study |
