# Supplementary material for: Regulation of arsenite oxidation by the phosphate two-component system PhoBR in Halomonas sp. HAL1
Source: Front Microbiol. 2015 Sep 9;6:923. doi: 10.3389/fmicb.2015.00923 (PMC4563254; doi:10.3389/fmicb.2015.00923)
Supplement: Supplementary file 2 [file Table2.DOCX]

**Table S2 Primers used in this study.**

| **Primer pairs** | **Primer sequences** | **Usage** |
| --- | --- | --- |
| pRLSR | AACAAGCCAGGGATGTAACG | For inverse PCR |
| pRLSF | CAGCAACACCTTCTTCACGA |  |
| phoB-up-F | AAAGACGTCGCTGGGCGGGGTATTT | For knockout of *phoB* |
| phoB-up-R | AAATGTACAGAACGGTTTTGGCGGTC |  |
| phoB-down-F | AAACCGCGGACCGGATACCGTTTCTCT | For knockout of *phoB* |
| phoB-down-R | AAAGAGCTCATCGTCACCGTACAGGG |  |
| phoB-yzF | CAGATAACGCCCAGGAT | For validation of HAL1-△*phoB* mutant strain |
| phoB-yzR | CGTAAGCGTCGAATATGC |  |
| phoB-upYZ-F | CTGGCTGCGTGGTGAAC | For validation of HAL1-△*phoB* mutant strain |
| phoB-upYZ-R | TGATGGTCGGAAGAGGC |  |
| phoB-downYZ-F | ATCTTCCCGACAACGC | For validation of HAL1-△*phoB* mutant strain |
| phoB-downYZ-R | TCTAACCGAGAGAGCAGC |  |
| PZori-dn | AAAATGCATTGCCACCTGACGTCTAAG | For construction of pCT-Zori |
| PZori-up | AAAATGCATGGTAACTGTCAGACCAAG |  |
| PhoB-HB-F | AAAGAGCTCGGTGGCATACGACACGCAATA | For complementation of *phoB* |
| PhoB-HB-R | AAAAAGCTTTTACAGGTATGGTGCGAGAGGTC |  |
| PhoB-F | AAAGGATCCATGACCGCCAAAACCG | For construction of PhoB protein over-expression vector using pET-28a |
| PhoB-R | AAAAAGCTTCATTTACACCCGTGCAGAG |  |
| PaioA-F | AGCAAACCTAAGCCTGAA(FAM labeling) | For EMSA of *aioBA* regulatory region DNA |
| PaioA-R | ACTGCTGCCGGTTAACT |  |
| Non-specific-F | ACTCGCCTCAAACACTCAAC (FAM labeling) | For EMSA of non-specific DNA |
| Non-specific-R | CTTTGGTCGTAAGACGCATA |  |
| PhoB-DZ-F | AAAGGATCCATGACCGCCAAAACCG | For construction of PhoB protein expression vector using pTRG |
| PhoB-DZ-R | AAAGAATTCCATTTACACCCGTGCAGAG |  |
| DZ-aioA-F | TCTAGAAGCAAACCTAAGCCTGAA | For bacterial one-hybrid experiment of *aioBA* regulatory region DNA |
| DZ-aioA-R | ACTGCTGCCGGTTAACT |  |
| Cm-F | AAAGGTACCTAACGACCCTGCCCTGAA | For construction of pCM184-Cm vector |
| Cm-R | AAAGGGCCCGTGTCCCTGTTGATACCG |  |
| pHaio-F | AAAAGATCTCTGGGAAAAAAACCAGAG | For construction of *aioBA*::*lacZ* |
| pHaio-R | AAATGTACAGTAGGGCAGGTTTGTGTT |  |
| placZ-F | AAATGTACAGCTATGACCATGATTACG | For construction of *aioBA*::*lacZ* or *phoBR*::*lacZ* |
| placZ-R | AAAGGTACCTAATGGATTTCCTTACGC |  |
| PphoB-F | AAAAGATCTATGGACGGTGGCATACGA | For construction of *phoBR*::*lacZ* |
| PphoB-R | AAATGTACAGGCGTTATCTGCTTCG |  |

* The underlined sequence denotes the restriction enzyme sites.
