# Supplementary material for: Regulation of arsenite oxidation by the phosphate two-component system PhoBR in Halomonas sp. HAL1
Source: Front Microbiol. 2015 Sep 9;6:923. doi: 10.3389/fmicb.2015.00923 (PMC4563254; doi:10.3389/fmicb.2015.00923)
Supplement: Supplementary file 3 [file Presentation1.PDF]

Construction of pCT-Zori and pCM-*lacZ*.

pCT-Zori was generated from a wide host, medium-copy vector pBHR1 (Szpirer et al., 2001) and a high-copy vector pGEM-4Z (Promega) (Fig. S1). The pBHR1 was digested with *Pst*I, while the fragment including the pUC replicon, multiple cloning sites and *lacZ*α gene was amplified from the vector pGEM-4Z using the primers PZori-up/PZori-dn (Table S2). The two fragments were then ligated via the isocaudomers *Pst*I and *Nsi*I, resulting in the new plasmid pCT-Zori, which is a wide host vector with the *lacZ*α selection marker and trans-conjunction function (Fig. S1). With plenty multiple cloning sites and the *lacZ*α selection marker, the pCT-Zori vector is more convenient for DNA cloning and the selection of transformants.

Because the mutant strains HAL1-$phoR_{931}$ and HAL1-△*phoB* both resist Kan, we replaced the Kan-resistant gene with the Cm-resistant gene in the allelic exchange vector pCM184 (Marx and Lidstrom, 2002) and constructed pCM184-Cm (see corresponding primers in Table S2) (Fig. S2). The complete *lacZ* coding region including the ATG codon was then amplified (primers listed in Table S2) and cloned into the *Bsr*GI-*Kpn*I sites of pCM184-Cm, generating a *lacZ* report vector pCM-*lacZ* (Fig. S2). The new *lacZ* report vector pCM-*lacZ* could insert the *lacZ* gene into the genome exactly between the tested gene and its regulation region, which could measure the expression level of the tested gene more precisely.

Over-expression and purification of PhoB.

The complete *phoB* coding region including the ATG start codon was PCR-cloned (primers listed in Table S2) as a *Bam*HI-*Hin*dIII fragment into double digested pET-28a(+) (Novegen) and then transformed into *E. coli* BL21 StarTM (DE3) pLysS (Invitrogen, Table S1). PhoB was over-expressed by adding 1 mM IPTG (isopropyl-β-D-thiogalactoside) when the OD_{600} reached 0.4. After 4 h of shaking cultivation at 28 ℃, the cultures were harvested by centrifugation at 13,400 g for 10 min at 4 ℃. After washing three times with buffer A (50 mM Tris-HCl, pH 8.0, 300 mM NaCl, 20 mM imidazole), the pellet was resuspended in 5 mL buffer A and lysed by sonication on ice for 5 min. After centrifugation, the soluble supernatant was

mixed with 1 mL pre-equilibrate Profinity^{TM} IMAC Resin (Bio-RAD) and gently agitated on ice for 2 h. The resin was then washed with five column volumes of buffer A followed by five column volumes of buffer B (50 mM Tris-HCl, pH 8.0, 300 mM NaCl, 40 mM imidazole). The target protein was eluted in 3 mL buffer C (50 mM Tris-HCl, pH 8.0, 300 mM NaCl, and 250 mM imidazole), dialyzed against buffer D (50 mM Tris-HCl, pH 8.0, and 300 mM NaCl) and detected with SDS-PAGE (Liu et al., 2012).
