# Supplementary material for: Regulation of arsenite oxidation by the phosphate two-component system PhoBR in Halomonas sp. HAL1
Source: Front Microbiol. 2015 Sep 9;6:923. doi: 10.3389/fmicb.2015.00923 (PMC4563254; doi:10.3389/fmicb.2015.00923)
Supplement: Supplementary file 4 [file Image1.PDF]

Chen et al., 2015

Figure S1

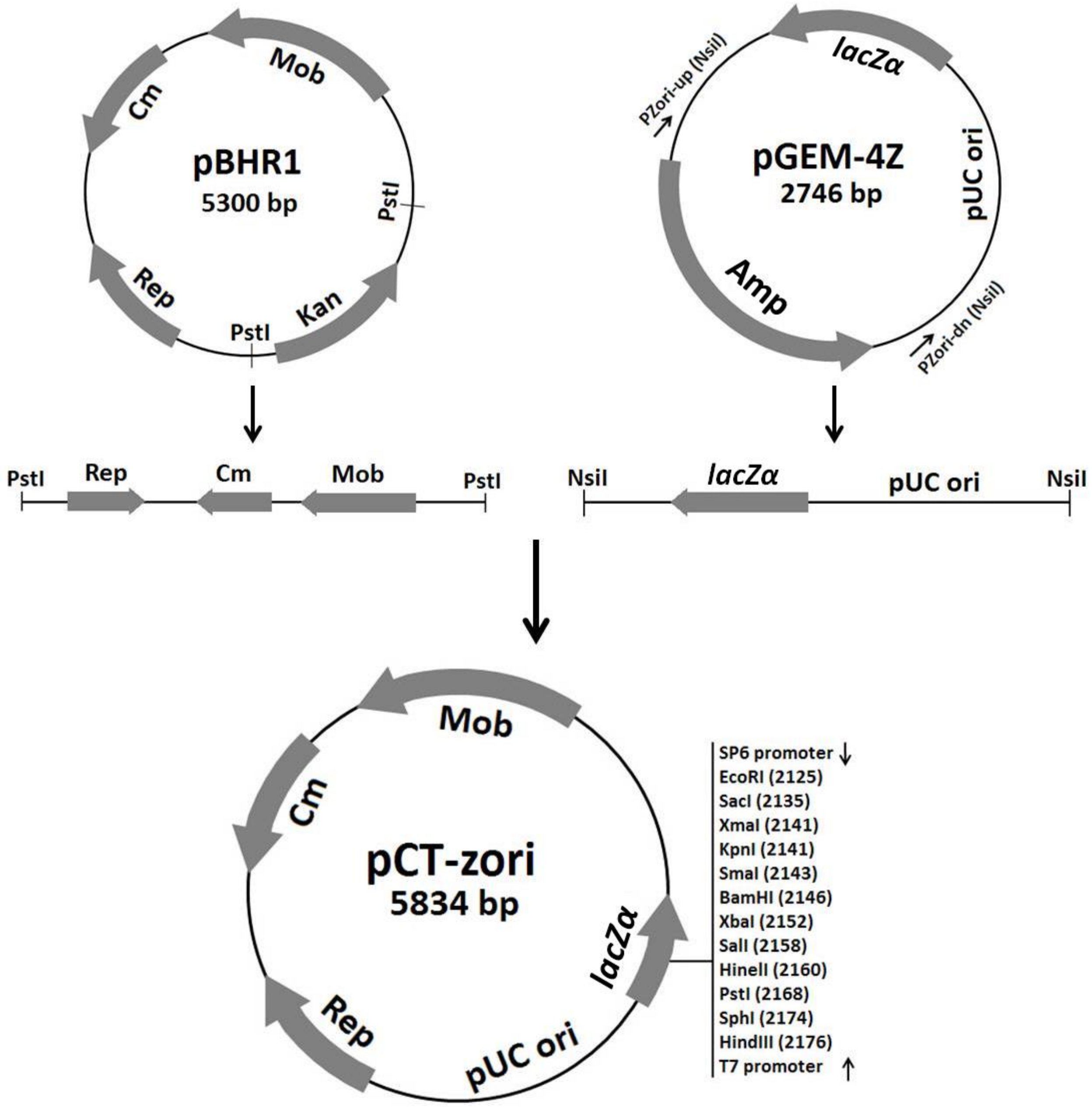

Fig. S1 The construction process and physical map of vector pCT-Zori. With two fragments from pBHR1 and pGEM-4Z, a wide host vector pCT-Zori with trans-conjunction function was constructed.
