# Supplementary material for: Regulation of arsenite oxidation by the phosphate two-component system PhoBR in Halomonas sp. HAL1
Source: Front Microbiol. 2015 Sep 9;6:923. doi: 10.3389/fmicb.2015.00923 (PMC4563254; doi:10.3389/fmicb.2015.00923)
Supplement: Supplementary file 5 [file Image2.PDF]

Chen et al., 2015

Figure S2

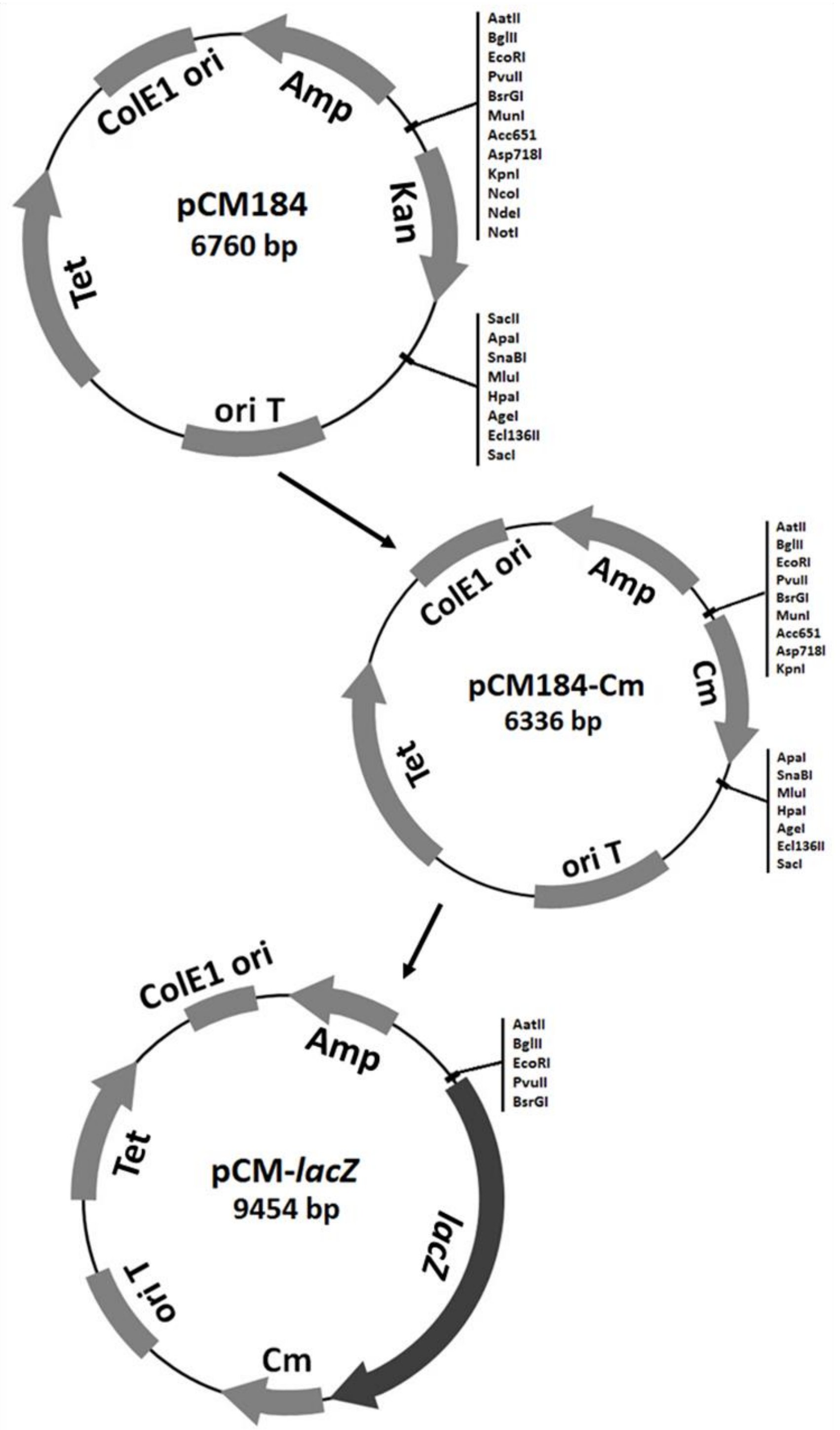

Fig. S2 The construction process and physical map of vector pCM-*lacZ*. The kanamycin resistance gene in low copy vector pCM184 was replaced by chloramphenicol resistance gene, resulting pCM184-Cm. Then the *lacZ* gene was inserted into *Bsr*GI-*Kpn*I sites of pCM184-Cm, yielding to the *lacZ* reporter vector pCM-*lacZ*.
