# Supplementary material for: Regulation of arsenite oxidation by the phosphate two-component system PhoBR in Halomonas sp. HAL1
Source: Front Microbiol. 2015 Sep 9;6:923. doi: 10.3389/fmicb.2015.00923 (PMC4563254; doi:10.3389/fmicb.2015.00923)
Supplement: Supplementary file 6 [file Image3.PDF]

Chen et al., 2015

Figure S3

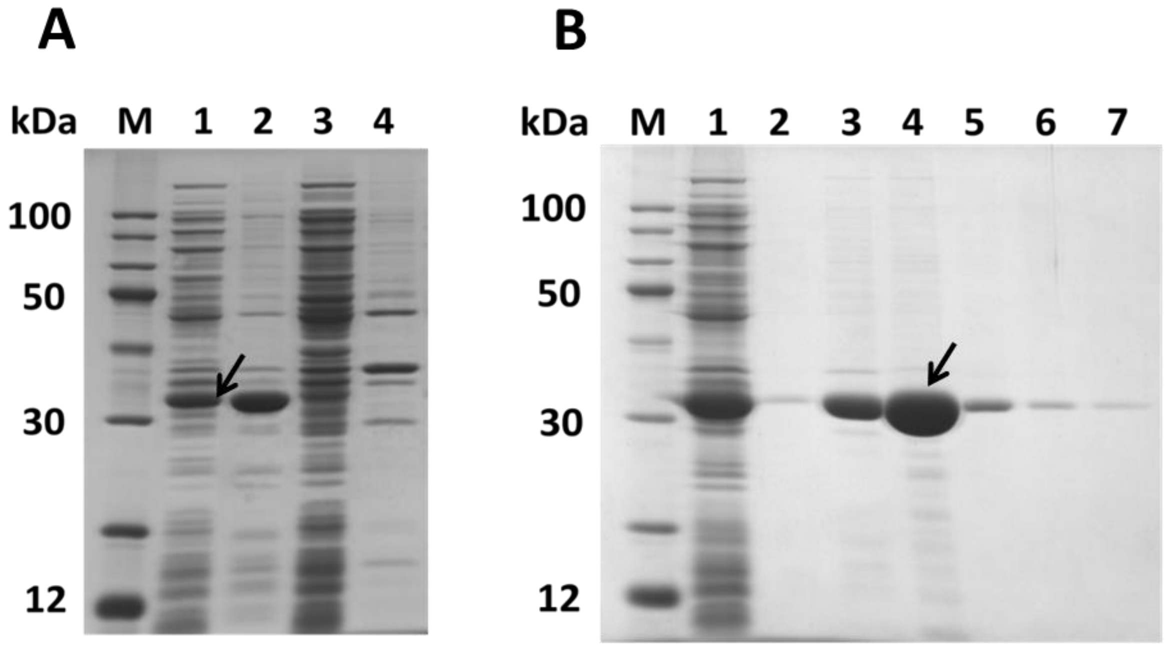

Fig. S3 Over-expression and purification of PhoB in *Halomonas* sp. HAL1. (A). The over-expression of PhoB with a His_6-tag in *E. coli* BL21. M, molecular weight standards; Lanes 1-2, supernatant and precipitate of total proteins in strain BL21-*phoB* induced by 1 mM IPTG for 4 h at 28 ℃, respectively. Lanes 3-4, supernatant and precipitate of total proteins in BL21-*phoB* without the IPTG induction. Arrow indicates the over-expressed PhoB. **(B)**. Purification of PhoB. M, molecular weight standards; Lane 1, the total proteins in strain BL21-*phoB*; Lanes 2-7, the eluted PhoB with 250 mM imidazole. Purified PhoB is indicated by arrow.
